# Radial arrangement of apical adhesive sites promotes contact self-alignment of fruits in *Commicarpus* plants (Nyctaginaceae)

**DOI:** 10.1038/s41598-017-10567-9

**Published:** 2017-09-08

**Authors:** Alexander E. Filippov, Elena V. Gorb, Stanislav N. Gorb

**Affiliations:** 1 Department of Functional Morphology and Biomechanics, Zoological Institute, Kiel University, Am Botanischen Garten 9, D-24118 Kiel, Germany; 2 Department N5, Donetsk Institute for Physics and Engineering, National Academy of Sciences of Ukraine, R. Luxemburg Str. 72, 83114 Donetsk, Ukraine

## Abstract

Fruits of the plants from the genus *Commicarpus* (Nyctaginaceae) use their adhesive properties for dispersal. They can readily stick to various surfaces including skin, fur, and feathers of potential dispersal vectors using the secretion provided by the set of glands arranged radially at the distal end of the cut-cone-shaped fruit. Field observations show that this particular geometry promotes self-alignment of the fruit to various surfaces after initial contact just by one gland is established. Such self-alignment in turn leads to an increase of the number of contacting points and to the enhancement of adhesive contact area. Here, we study this particular geometry from a theoretical point of view, by probing adhesion ability of geometries having from 2 to 7 radially distributed attachment points. The results show that the radial arrangement provides rapid alignment to the surface. The robust adhesion can be reached already at 5 adhesive points and their further increase does not substantially improve the performance. This study is important not only for our understanding of the functional morphology of biological adhesive systems, but also for the development of technical self-aligning adhesive devices.

## Introduction

**Figure 1:**
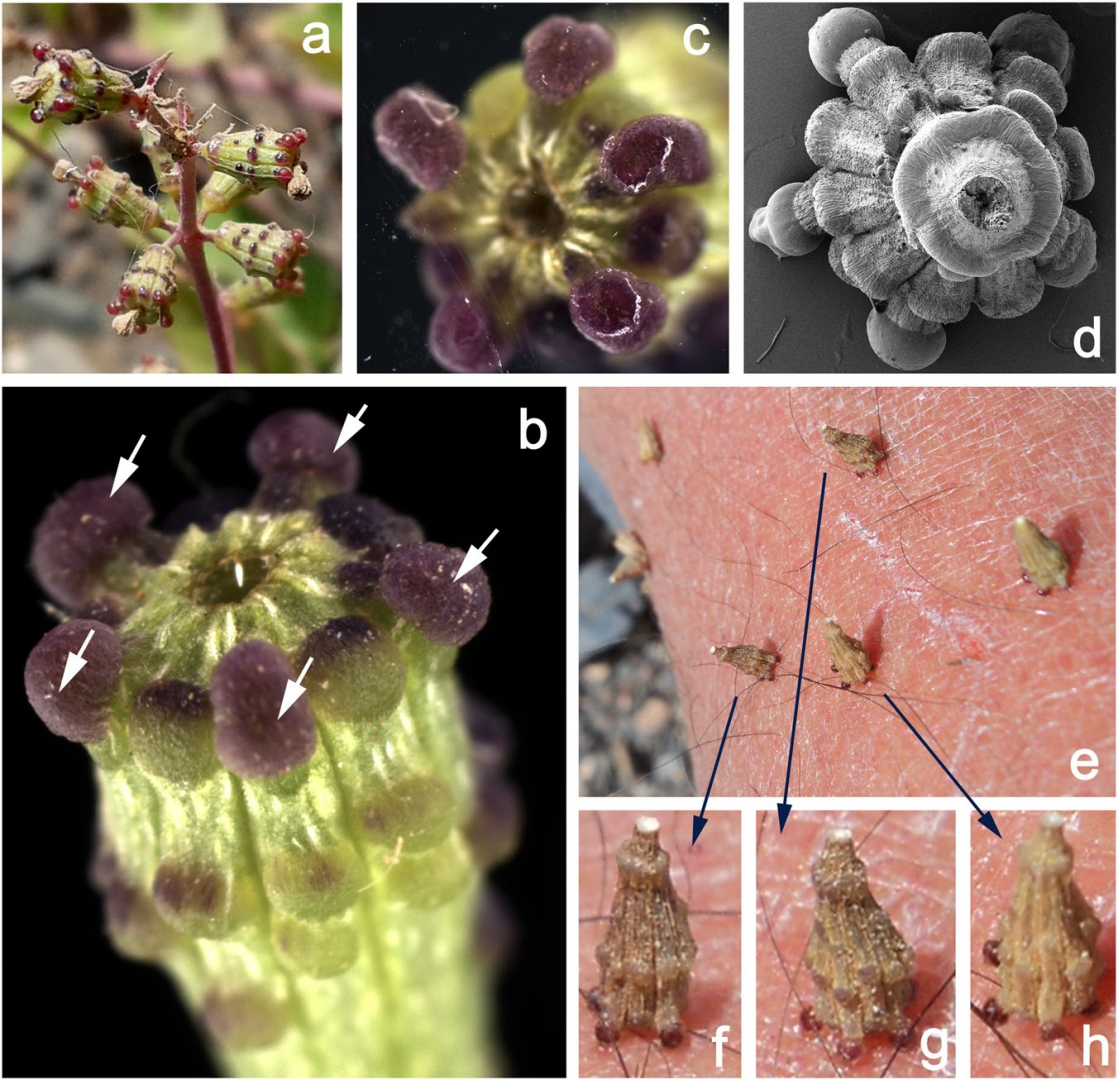
Fruits (anthocarps) of the *Commicarpus helenas* plant. (**a**) Intact fruits on the plant ready for adhesion to the surface of a potential dispersal vector. (**b**) Single fruit with five radially arranged apical glands (marked here with arrows) delivering adhesive secretion as soon as contact is formed. (**c**). Single not completely aligned fruit adhering with only two glands (glands on the right hand side) to the glass surface (view from below through the transparent glass). (**d**). Single completely aligned fruit adhering with five glands to the glass surface (view from above, cryo-SEM image). (**e**–**h**) Field situation: fruits adhering to the human skin. Please, note that most of them adhere to the uneven and rather corrugated surface with all five radially arranged apical glands.

Genus *Commicarpus* consists of about 30–35 species distributed in arid areas of Africa and western Asia^1, 2^. Fruit structure is 10-ribbed with 5–10 viscid and 5 mucilaginous glands at the distal part of the funnel-shaped fruit^3^. As described by Struwig and Siebert^3^, after fertilization, the upper, petaloid part of the flower falls off, whereas the lower part enlarges and develops into a protective structure around the fruit, which is collectively called the *anthocarp* ^4–6^ (Fig. 1a). The shape of the anthocarp and the arrangement of glands are species specific for *Commicarpus* ^7, 8^. The shape of the anthocarps varies in different species from cylindrical, fusiform, clavate to elliptic clavate. The apex is surrounded by either 5 or 10 glands, which can be stalked or sessile (Fig. 1b). Additionally, sessile wart-like glands are scattered across the surface of the anthrocarp (called fruit throughout the text) below the apex (Fig. 1b).

In ripe fruits, when an apical gland touches the surface of a moving object, the adhesive secretion is discharged and the fruit generates an adhesive contact with the surface (Fig. 1c). When the pulling force is further applied, the ripe fruit can be easily detached from the plant stem and remain adhering to the substrate (Fig. 1d). Our field observations showed that the applied pulling force to the fruit, which has built an initial contact with one apical gland, effected in a self-alignment of the fruit on the substrate and this led to the situation, when additional apical glands were coming into contact (Fig. 1e–h). The higher number of glands provided stronger adhesion to the surface. Since radial arrangement of 5–10 apical adhesive glands is very characteristic for the *Commicarpus* fruits, one can ask, whether the specific shape of the fruit and radial arrangement of apical glands are adaptations for the self-alignment and adhesion enhancement during the initial contact of the fruit with a potential dispersal vector.

We observed *Commicarpus helenas* plants at Fuerteventura (Canary Islands, Spain)^9^ and realized their quick and robust adhesive contact formation with almost any surface independently of its structure and chemistry. More than 80% of fruits have been observed adhered by the full set of apical adhesive glands also to uneven and corrugated surfaces. In the present study, we applied numerical modeling to answer following questions. (1) Does specific shape of the fruit and the specific apical+radial arrangement of adhesive glands provide self-alignment mechanism to various substrate geometries during contact formation? (2) What is the minimal/optimal number of apical glands necessary for contact formation? (3) Is there any dependence of the self-alignment effectiveness on the direction of the external pulling force relatively to the position of the first gland in contact?

## Numerical model and simulations

**Figure 2:**
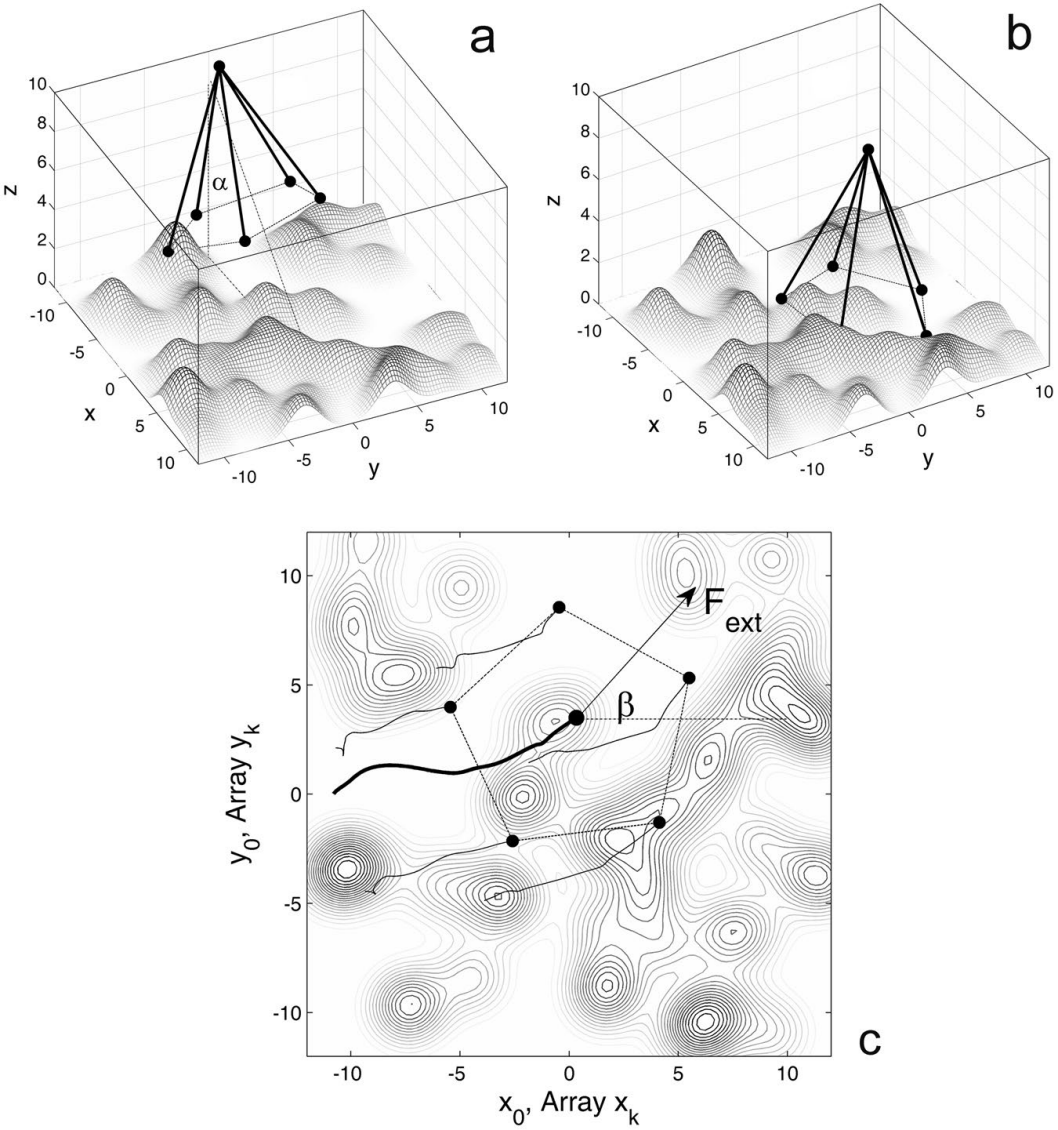
Conceptual structure of the model (**a,b**) and vertical projection of the system (**c**). Subplot (**a**) illustrates the pyramid with *N* apical contact points. In this particular case, *N* = 5 corresponds to the real configuration of the *Commicarpus* fruit. The pyramid is originally placed at the angle *α* to the vertical line of the pyramid near rough substrate surface. It is attracted to the surface by apical contact points corresponding to the apical glands of the real fruit and at some intermediate time moment, it is turned to contact with the surface, depending on the relative configuration of apical points to the surface profile, as shown in subplot (**b**). Subplot (**c**) illustrates the projections of the trajectories of apical points (glands) and the central point, plotted by the thin and bold curves, respectively, between time moments shown in the subplots (**a**) and (**b**). The contour plot reproduces the same realization of the random rough surface as represented in the subplots (**a**) and (**b**). Constant external force *F*_*ext*_ is applied to the central point of the pyramid at the azimuth angle *β* to the axis *x*. All the distances here are normalized to the size of the glands *﻿r*_0_ = 1 (which is equal to ﻿*r*_0_ = 500 μm in physical units).

To simulate numerically adhesive contact of the radially arranged apical glands, we constructed the following simplified model (Fig. 2a,b). First of all, we reduced a complex structure of the fruit. The shape of the natural system has almost rotational cylindrical symmetry (or maybe rather close to a conical structure) with some number of adhesive contact glands. However, to simplify the model we will reduce the structure to a kind of “pyramid” having *N* adhesive contact points and one central vortex, which are elastically connected one with another. The cylindrical symmetry will be reflected in the model by the isotropical connection between every contact point with its nearest neighbors as well as with the center of the circle in the pyramid base and with the main vortex on its top.

The main simplification here is a reduction of number of degrees of freedom. Instead of a continuous surface we conserve only discrete number of main elements and connections between them. It allows us to strongly reduce time consuming calculations. Let us note also that in the simulation, the number of the contact glands *N* = 2, 3, ... is not restricted by actual experimental observations. It will be varied to allow us to model different systems, which even do not exist in nature, in order to extract the information for further possible optimization.

In the particular case illustrated in Fig. 2a,b, we used the same number *N* = 5 as in the real system depicted in Fig. 1. We also accounted that before contact formation in initially non-contact state near rough surface, the pyramid is placed at some arbitrary angle *α* to the vertical line: such a configuration is reproduced in Fig. 2a.

It is supposed that due to adhesion force, an apical gland of the fruit being close enough to the surface is generally attracted to the substrate. However, the adhesion is a short range force and the pyramid, even contacting with the surface by one or two of its possible contact points (apical glands), can remain for a long time attached by the only these initially contacting points. The principle possibility, rate of the attraction process and stability of the final completely “attached” configuration is determined by a compromise between a number of competing forces and parameters of the system/problem. Some of these parameters are (1) roughness of the surface, (2) adhesive strength generated by the glands, (3) external force acting on the fruit, (4) angle, at which the external force is applied, and (5) its fluctuations with the time.

To study the relative role of different parameters in the fruit alignment and to avoid too time consuming calculations, we minimized a number of mechanical degrees of freedom of the problem to as small as possible and reproduced the system by the only *N* + 1 movable points, where *n* = 1, 2, ..., *N* basal adhesive points are elastically connected with each other as well as with the one in the geometrical center of the apical construction. The latter point is further numerated as the point number zero *n* = 0.

The real fruit is relatively rigid, but the apical glands due to the adhesive layer are slightly flexible. To maintain more or less constant shape of the structure with very few movable segments in three-dimensional space, the elastic interaction can be organized as it was done in previous studies (see for review Popov *et al*.^10^ and references there). It is provided with strong longitudinal stiffness of 2-valley potential *k* ^||^ preventing extension and compression of practically rigid segments connecting the neighboring nodes with each other ${\vec{r}}_{j}$ and with the central one (*j* = 0, 1, ..., *N*). The corresponding force ${\overrightarrow{f}}_{jk}^{||}={k}^{||}({\overrightarrow{r}}_{j}-{\overrightarrow{r}}_{k})[1-{(({\overrightarrow{r}}_{j}-{\overrightarrow{r}}_{k})/{r}_{0jk})}^{2}]$ tends to keep a distance between the nodes ${\vec{r}}_{j}$ and ${\vec{r}}_{k}$ close to the equilibrium *r*_0*jk*_ due to the attraction factor $[1-{(({\vec{r}}_{j}-{\vec{r}}_{k})/{r}_{0jk})}^{2}]$. The equilibrium array *r*_0*jk*_ is calculated from the trial initial distribution of the segments reproducing the realistic configuration.

For definiteness and simplicity, the adhesion force is modeled by the Morse potential ${U}_{VdW}=$ ${f}_{0}{r}_{0}[1-\exp {(-(r-{r}_{0})/{r}_{0})}^{2}]$, which is often used in such studies. From preliminary experiments, we have estimations for the physical values of the parameters *f*_0_ and *r*_0_. The characteristic force *f*_0_ belongs to the interval 65 mN ≤ *f*_0_ ≤ 71 mN and the distance *r*_0_ of effective adhesive interaction ${U}_{VdW}={f}_{0}{r}_{0}[1-\exp {(-(r-{r}_{0})/{r}_{0})}^{2}]$ should be associated with the real size of the glands *r*_0_ = 500 μm.

It is convenient to perform all the calculations in dimensionless units. Below, all the lengths and forces of the problem are measured in the units of the effective adhesion strength *f*_0_ and its characteristic distance *r*_0_, respectively. In other words, below *f*_0_ = 1 and *r*_0_ = 1. In the same units, the height *H* of the cone (pyramid) is equal to *H* ≈ 10*r*_0_ = 10 and the radius of the circle in its base *R* is approximately: *R* ≈ 5.8*r*_0_ = 5.8 (independently on the number of adhesive points placed on its perimeter).

Equilibrium distances *r*_0*jk*_ between the glands and vortex of the pyramide are completely defined by the geometry. They should be numerically recalculated each time for every particular number of the glands *N* = 2, 3, ... . For example, the distance between the nearest neighbors on the pentagonal *N* = 5 base is equal to *r*_0*jj* + 1_ =$R\sqrt{(5-\sqrt{5})/2}\approx 6.8$.

Another important value is the longitudinal stiffness *k* ^||^ of 2-valley potential in the formula ${\vec{f}}_{jk}^{||}={k}^{||}({\vec{r}}_{j}-{\vec{r}}_{k})[1-{(({\vec{r}}_{j}-{\vec{r}}_{k})/{r}_{0jk})}^{2}]$. It can be also recalculated now in dimensionless form. Young’s modulus *E* of the seed measured experimentally by us is in average about 8 GPa. Being now applied to the elastic interaction between the glands ${k}^{||}=\frac{{r}_{0}^{2}E}{{f}_{0}({r}_{0jj+1}/{r}_{0})}$ and normalized to *f*_0_ and *r*_0_ in physical units, it gives dimensionless coefficient *k* ^||^ approximately equal to *k* ^||^ ≈ 9.8. This value makes the pyramid quite rigid, but still allows small deformations in the frames of the model. These deformations are slightly visible in the short projections of the trajectories of the contact points to the surface in Fig. 2.

The rough surface *Z*(*x*, *y*) can be alternatively modeled either as a sum of Fourier harmonics $Z(x)=\frac{1}{2\pi }{\int }_{{q}_{\min }}^{{q}_{\max }}c(q)\cos (qx+\xi )$ with scaling spectrum *c*(*q*) = *c*_0_ *q* ^*β*^ and random δ-correlated phase 〈*ζ*(*x*)〉 = 0, 〈*ζ*(*x*)*ζ*(*x*′)〉 = *δ*(*x* − *x*′) (see Popov *et al*.^10^) or by a random deposition of localized functions (Gaussians, for example) with randomly varied positions, amplitudes and widths: $Z(x,y)=\sum _{n}{G}_{n}(x,y)=\sum _{n}{a}_{n}\exp [-({(x-{x}_{n})}^{2}+{(y-{y}_{n})}^{2})/{w}_{n}^{2}]$. In the second case, the structure is regulated by a number of Gaussians, their widths and typical distance between the hills and valleys of the desired randomly accumulated surface *Z*(*x*, *y*) = ∑_*n*_ *G*_*n*_.

In both cases, the limiting scales of the surface irregularities are defined by a choice of the smallest and largest wavelengths regulated by *q*_max_ and *q*_min_ in Fourier approach, or by the minimal *w*_min_ and maximal *w*_max_ widths of the Gaussians included into the expansions. For some biological applications besides to ordinary fractal component, a contact substrate can include also well pronounced regular superstructure with its own characteristic scale. In such a case, the second variant seems to be more convenient, because it allows naturally including it to the expansion or simply enhancing the corresponding scale in it.

From biological point of view, it reminds well known and already previously studied by us experimental situations with the spatules of different animals^11^. The spatules practically ignore irregularities, which are much smaller or much larger than their own size. As a result, they effectively “feel” such surfaces as almost flat.

Additional reason for this choice appears from limitations of the minimalistic models like the one, which is used here. Minimal and maximal scales of roughness are limited by the size of solitary gland (radius of the adhesion *r*_0_ in the model) and the object size itself (height *H* and radius *R* of the pyramid, which are only 6÷10 times bigger than *r*_0_). So, the model pyramid simply cannot penetrate deeper than *r*_0_ and cannot overcome irregularities bigger than its own height *H* = 10*r*_0_.

It dictates the limitations that the scales of the irregularities have to be comparable with the scales of the pyramid itself. Finally, the amplitude of roughness after accumulation is regulated by the normalization $Z(x,y)\to A(Z(x,y)-\,\min (Z))/(\max (Z)-\,\min (Z))$, where a desirable amplitude *A* can be chosen from the limit of the flat surface *A* = 0 to the values comparable with the lengths of the pyramid segments.

The restriction that the widths of the Gaussians are limited by *w*_min_ comparable with *r*_0_ does not mean that the pyramid does not interact completely with the smaller scales. First of all, it is supposed that every apical point (gland) interacts by adhesion force with each segment of the numerically generated discrete array of the surface *Z*(*x*, *y*) with much smaller cells of the array. Besides, an interaction with the smaller scales is included in the model by the dissipation, which phenomenologically involves all the losses down to the very small scales, like microscopic excitations inside the surface.

The central vortex on top of the pyramid (which is not adhesive) is affected by the constant external force ${\vec{F}}_{ext}$. In general case, this force can be directed at some (quite arbitrary) angle *β* to the vertical plane (*x*, *z*). Due to elastic connection between this point and all other ones, the force is transferred to the motion of the whole pyramid.

It is generally accepted that for the scales of the problem under consideration, one can neglect inertial terms in Newtonian equations and reduce the problem into an over-damped one. Then motion takes the following form:

$$\tau \,\partial {\vec{r}}_{n}/\partial t={\vec{f}}_{elastic}+{\vec{f}}_{VdW}+{\vec{F}}_{ext}.$$

Here, the forces have different nature depending on the particular point with the numbers *n* = 0 and *n* = 1, .., *N* and must be numerically accumulated from all the sources described above. As typical for over-damped equations, the multiplicative constant *τ* defines characteristic time of the process and can be used as a unit to measure all time-dependant values: *τ* = 1.

In a general case, being affected by the combined adhesion, elastic and external forces, the pyramid is moving and rotating in three-dimensional space. Adhesion gradually turns its basal plane to the surface and at the same time, the system tends to shift itself along the substrate in a some direction found as a compromise between the relief of *U*_*VdW*_ and the external force ${\vec{F}}_{ext}$. A projection of this motion to the horizontal plane (*x*, *y*) for the same particular realization of the surface *Z*(*x*, *y*) = ∑_*n*_ *G*_*n*_ as in Fig. 2a,b is reproduced in Fig. 2c.

Intuitively, one can expect that if the external force is weak enough, the pyramid would fall by its base to the surface, stick to it due to the adhesion force and stop. In an opposite limit, the strong force pulling the pyramid top would overcome adhesion and continue to rotate the system in spite of some instant contact of its basal points to the surface. So, even after some temporal contact, the point (gland) can be detached from the surface.

It is important to admit that the present simple model completely ignores fine processes lying under the adhesion in tangential contact, in particular, a chemical process of destruction and recovering of the contact after its destruction. However, our preliminary experimental observations demonstrate that a full recovery of adhesive contact in multiple bonding and debonding cycles is possible. The aim of our modeling was only to get insight into a very specific shape of the fruit and particular distribution of its adhesive sites. The simple model describes its self-alignment properties very well, and that is why the more complex models are here superfluous.

**Figure 3:**
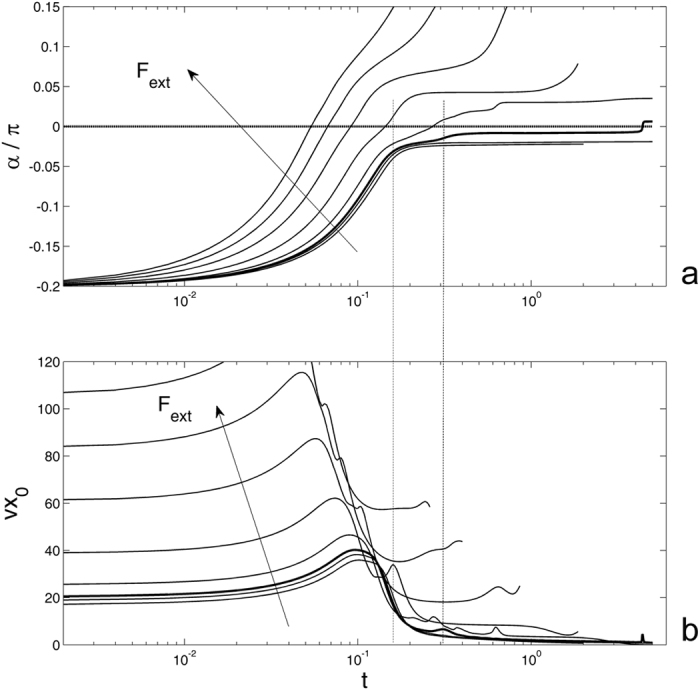
Time-dependant angle *α* (**a**) and the velocity of the central point *Vx*_0_ (**b**) calculated at different values of the external force for the flat contact surface. The saddle-point curve corresponding to the critical value of the force *F*_*ext*_, at which the system stops exactly in the vertical position, is plotted by the bold line. For more explanations, see the text.

Let us return back to the statement that if the external force is weak enough, the pyramid falls to the surface and stops, but if the force is strong, some of the contacts detach again and the pyramid restarts moving. This qualitative observation can be quantitatively supported by a calculation of the time dependencies for the angle *α*(*t*) and velocity of the central point ${\vec{V}}_{0}(t)$. To reduce the number of degrees of freedom, we first study the flat surface *A* = 0 and motion along *x*-axis only at *β* = 0 and ${\vec{V}}_{0}(t) \rightarrow V{x}_{0}(t)$. The results of this study are accumulated in Fig. 3, where time-dependant angle *α* and the velocity of the central point *Vx*_0_ are calculated at different values of the external force. Here, the curves are plotted in logarithmic scale along the time axis in order to extend short time interval of fast rotation at the very beginning and to compress much longer asymptotic part with small changes of the variables. The main reason for this difference in the rates of the process is related to a strong impact to the motion of the central point caused by the rotation of the pyramid. This fact is clearly seen from the comparison of the subplots (a) and (b) in Fig. 3. Even small deflections in the curves for the angle *α* = *α*(*t*) are accompanied by the maximums of the velocity *Vx*_0_(*t*). As an example, two local maxima of *Vx*_0_(*t*) are marked by the vertical lines crossing both subplots in Fig. 3.

**Figure 4:**
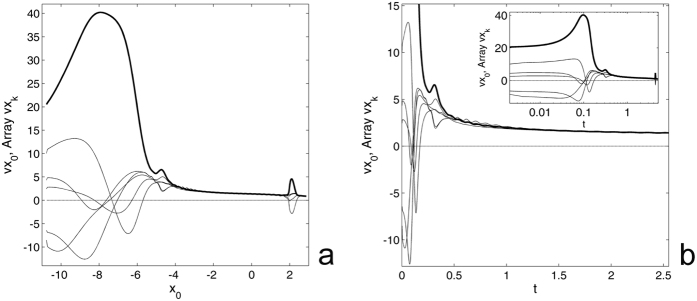
Spatial- and time-dependencies of the velocities. Subplots (**a**) and (**b**) show the velocities of the central point (bold line) and array of the contact points *vx*_*k*_ (where *k* = 1, 2, .., *N*), respectively. The insert shows the same time-dependence as the main subplot (**b**) in logarithmic scale in order to extend an initial interval of fast motion at the beginning of the process.

Rotation of the pyramid is also accompanied by relatively fast motions of all adhesive points. This motion is presented in Fig. 4 by a family of thin curves for the array *vx*_*k*_(*t*), where *k* = 1, 2, ..., *N*. The maximums of these curves are much lower that the main maximum of *Vx*_0_(*t*) (bold line), but still well pronounced. It is also important to note a strong difference between coordinate- and time-dependencies of the velocities in subplots (a) and (b), respectively. The reason for this is that the displacements along the *x*-axis stop quite quickly. This means that the contact points are almost completely attached to the substrate and the variable *x*_0_ practically does not change after *x*_0_ ≈ 2. However, some slow drift of all components of the system at longer time still occurs. It can be seen in the logarithmic plot shown in the insert to the subplot (b).

As for the situation with an arbitrary orientation of the external force *β* ≠ 0, counting that the pyramid starts to build contact from arbitrary inclined position with non-zero angle *α* ≠ 0, one can expect that its rotation will essentially depend on the direction of the external force. Indeed, when an external influence is absent (*F*_*ext*_ = 0), the pyramid spontaneously falls to the surface into the direction of *x*. If the external force (*F*_*ext*_ ≠ 0) is parallel to this direction, it supports the rotation. In all other cases, the result of interplay between this force and adhesion is not so transparent and one needs to perform the further numerical experiment.

**Figure 5:**
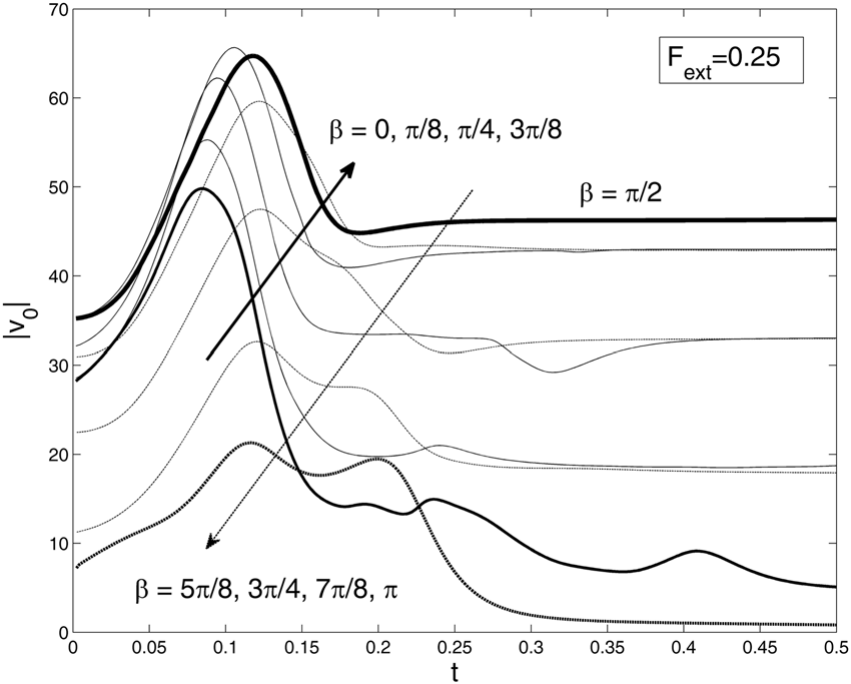
Azimuth angle *β* dependence of the time-dependant absolute velocity of the central point |*v*_0_| at the fixed intermediate external force *F*_*ext*_ = 0.25. The curves corresponding to the symmetrically important directions *β* = 0,*π*/2,*π* are plotted by the bold lines.

The results of the solution for a number of different angles $\beta =0,\,\pi /8,\,\pi /4,\,\mathrm{.}.\,\pi$ are collected in Fig. 5. It is demonstrated that up to the perpendicular force *β* = *π*/2, an absolute velocity $|{V}_{0}|=\sqrt{V{{x}_{0}}^{2}+V{{y}_{0}}^{2}}$ generally increases: this tendency is shown by the bold arrow in Fig. 5. After this value, the tendency changes to the reverse one and is shown by the opposite, fine arrow in the same Fig. 5. The reason for this is that the *x*-projection of the external force is now opposite to the *x*-projection of the adhesion force. These two forces compete at the beginning of the process and reduce the pyramid rotation and, as a result, reduce total velocity |*V*_0_|. It is found that the minimum of horizontal velocity is reached for exactly negative orientation of the external force at *β* = *π*.

**Figure 6:**
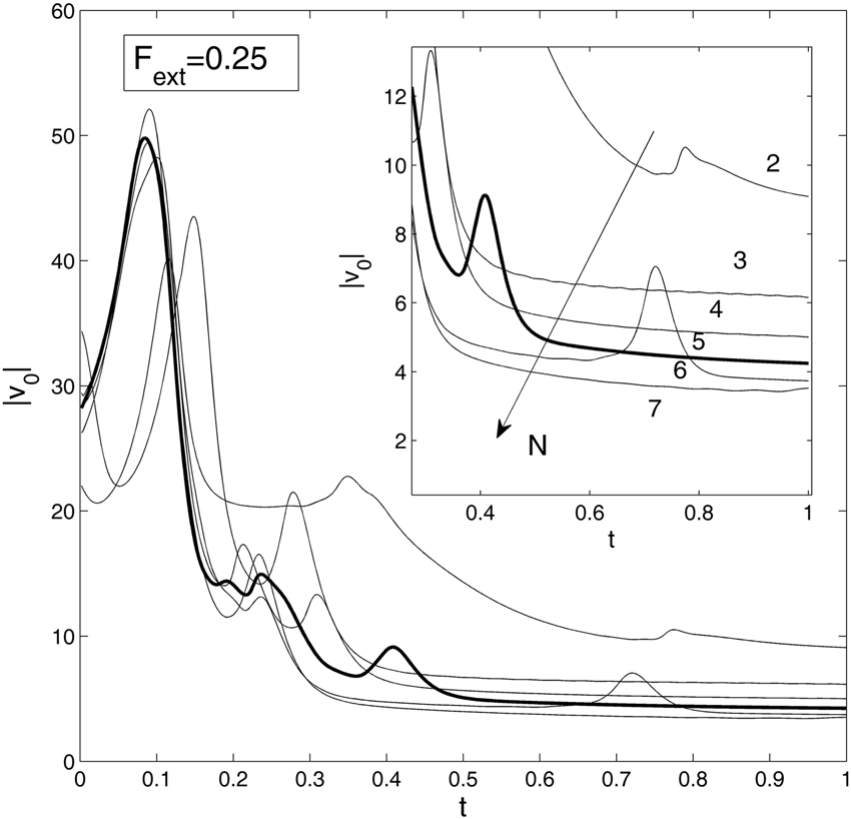
Time-dependant absolute velocity |*v*_0_| at different numbers of contact points *N* = 2, 3, 4, ... . Inset magnifies the |*v*_0_| values for the range of 0.3 < t < 1, to illustrate monotonous tendency of the velocity |*v*_0_| with the number *N*.

To discuss the minimal/optimal number of apical glands necessary for contact formation, we now examine the simplest case of the fixed angle *β* = 0, the value of the external force *F*_*ext*_ = 0.25, and vary the number of glands *N* = 2, 3, 4, ... . Figure 6 presents the results of the simulation based on these constants. Here, the family of time-dependant absolute velocities |*v*_0_| at different number of the contacts *N* = 2, 3, 4, ... is generated. From the main plot of this figure, we see that for the numbers *N* = 5, *N* = 6 and *N* = 7, the curves practically coincide at starting short time intervals. Small differences appear for the longer times. The insert in Fig. 6, which magnifies vertical scale for a fragment of the full image, illustrates monotonous variation of |*v*_0_| for different values of *N*.

Above, we artificially reduced roughness of the substrate to the zero *A* = 0 in order to make the dependencies on different parameters of the problem regular and more transparent. However, for any contact problem, the amplitude of roughness is a very important property of the real surface. To extract information related to this property in the same regular manner, as we did this before for other parameters, we return now to the fixed number of the glands and chose again *N* = 5, as it represents the most interesting biological case. Besides, we return to the trivial angle *β* = 0 and again keep the fixed intermediate value of the external force *F*_*ext*_ = 0.25.

It is important to note that the chosen value *F*_*ext*_ = 0.25 gave a possibility to study all stages of fruit motion in each numerical experiment. This force is not too strong to immediately turn the fruit (and quickly remove it from contact), but not too weak to allow the apical glands to stick to the substrate from the very beginning of the contact formation. Intuitively, one can expect that varying roughness of the contact surface *A* ≠ 0 would cause a competition of these two factors: From one side, if the surface is strongly corrugated, it is difficult to adjust a good spatial configuration of the glands for simultaneous contact of all or the majority of them with the surface. From the other side, if the surface consists of deep “hills and valleys”, every gland being once attached can be strongly trapped in narrow spaces between hills of the roughness. In this case, it may be difficult to remove it from such a trapped position.

Which factor will prevail in the contact formation process certainly depends on the spatial distribution of the contacts. One can expect that simple planar “contact pad” can conflict with the random asperities on a complex surface. First occasionally formed contact will prevent formation of new ones. From our previous studies of the analogous contact problems, we know that adhesion can be strongly enhanced by the “hairy” configuration of the contact device^10^, which allows every fragment of the system to turn and find more optimal contact configuration. In order to test this idea for the adhesive fruit system under consideration, we calculate the time-dependant angle *α*(*t*) and absolute velocity |*v*_0_| for a set of different amplitudes of the roughness *A*.

**Figure 7:**
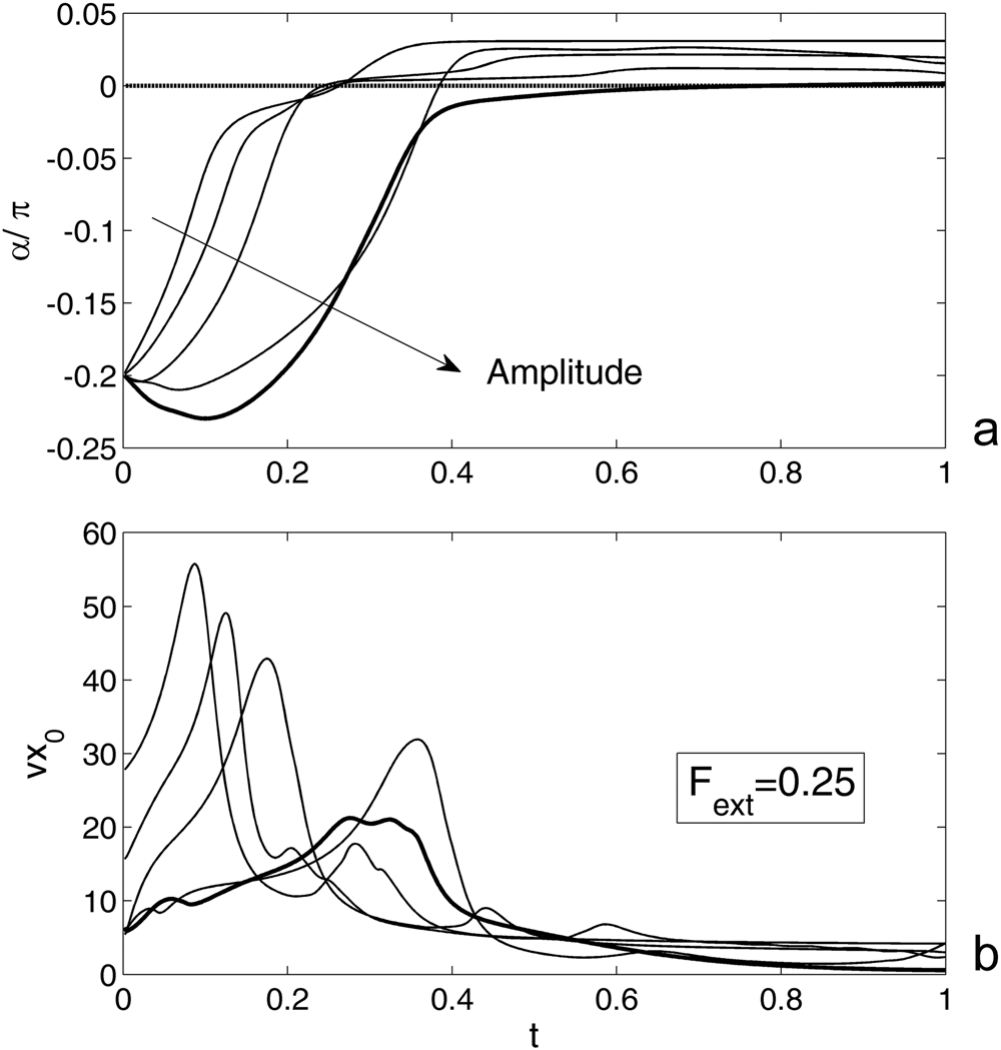
Time-dependant angle *α* (**a**) and absolute velocity |*v*_0_| (**b**) at different amplitudes of the roughness. *A* = 0, 0.125, 0.25, 0.375, 0.5 varies from the completely flat surface *A* = 0 to the roughness *A*_*crit*_ ≈ 0.5, at which external force *F*_*ext*_ = 0.25 becomes critical; corresponding curves are marked by the bold lines.

Figure 7 shows that despite of a relatively complex behavior, the contact ability of the adhesive fruit system is generally stronger on rough substrates. The value *A* = 0, 0.125, 0.25, 0.375, 0.5 varies starting from the completely flat surface *A* = 0 up to the new critical one *A*_*crit*_ ≈ 0.5. The latter value corresponds to the roughness, at which previously “intermediate” external force *F*_*ext*_ = 0.25 becomes critical. This means that in contrast to the flat substrate surface at *F*_*ext*_ = 0.25, the system completely adheres to the substrate. Both the angle *α* and absolute velocity |*v*_0_| simultaneously tend to zero: corresponding curves in Fig. 7a and b are marked by the bold lines.

In reality, another important source for randomness and possible instability is the randomly fluctuating external force *F*_*ext*_. Certainly, we can not account for all the possible sources of the fluctuations, but it can be assumed that the force *F*_*ext*_, caused by potential dispersal agents, such as vertebrates, is never fixed in a real situation. Additionally, the angle *β* of the force acting on the fruit is continuously varying. Below, we model the second reason of the fluctuations.

Mathematically, these fluctuations mean that even at more or less fixed strength of the external force *F*_*ext*_ ≈ *const*., the direction of the force *F*_*ext*_ (for example, caused by motion of animals, or by hits of some close branches in the bush perturbed by wind, etc.) permanently changes. To simulate this, one requires an additional equation (providing a kind of chaotic component) for the angle ∂*β*/∂*t* = *ξ*(*t*) to the equations of motion. It describes so-called „random walk“ of the angle *β*. Time-dependant source of the fluctuations *ξ*(*t*) here is *δ*-correlated Gaussian noise with zero mean value 〈*ξ*(*t*)〉 = 0 and the strength 〈*ξ*(*t*)*ξ*(*t*')〉 = *σδ*(*t* − *t*'), which is defined by the intensity *σ* of the fluctuations.

Above (see Fig. 5), we already saw that different angles *β* led to different dynamic scenarios changing the absolute velocity |*V*_0_| in particular time dependence. Now, the angle *β* of the external force *F*_*ext*_ permanently changes ∂*β*/∂*t* = *ξ*(*t*). Depending on the relationship between characteristic times and forces of the attachment from one side and fluctuations from another side, different scenarios are possible.

**Figure 8:**
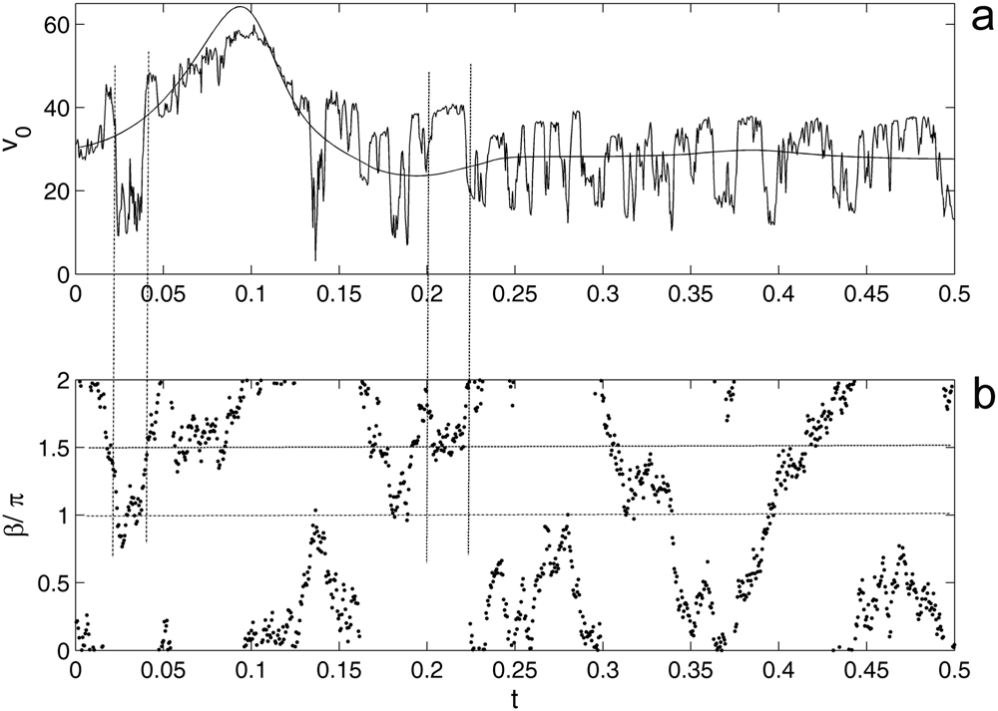
Time-dependant absolute velocity |*v*_0_| at random walk of the azimuth angle *β*. The rotation of the angle *β*(*t*) in the plot is reduced to the physical interval [0,2*π*]. The vertical straight lines mark two typical regions, where the angle is close to the values of maximal and minimal velocities (near *β* = *π* and *β* = 3*π*/2, respectively).

If the fluctuations are too weak (or if their oscillations are too fast), they will not affect the motion at all. In the opposite limit of sufficient force *F*_*ext*_ and slow rotation of its direction ∂*β*/∂*t* = *ξ*(*t*), we return to already discussed case of the strong and regular force *F*_*ext*_. The most interesting intermediate case is presented in Fig. 8, where complicated time dependency is shown. It corresponds to the situation, when the direction of *F*_*ext*_ changes few times during characteristic time of rotation of the angle *α* and, as a result, absolute velocity |*V*_0_|.

One can see that despite extremely strong effect of fluctuations in this case and visually wide variations of the velocity |*V*_0_|, the behavior remains stable and the fruit after all attaches to the surface. It is interesting to note here well pronounced correlations between the instant angles *β* in Fig. 8b and resulting velocities |*V*_0_| in Fig. 8a. These correlations are found to be in perfect agreement with the results found earlier for the array of regular fixed angles *β* presented in Fig. 5.

It is expected from Fig. 5 that when randomly walking angle *β*(*t*) for relatively long time remains near the values close to *β* = *π* or *β* = *π*/2 (*β* = 3*π*/2), the velocity would attract to its maximal or minimal values at this stage of motion. To elucidate this, we plot the rotation of the angle *β*(*t*) in Fig. 8b as reduced to the physical interval [0,2*π*]. Two typical regions, where the angle is close to the values of maximal and minimal velocities (near *β* = *π* and *β* = 3*π*/2, respectively), are marked by the vertical straight lines connecting both subplots.

Desirable stability of the behavior even under relatively strong fluctuation means that despite of strong variations, absolute velocity |*V*_0_| still remains close to the function obtained before at an intermediate constant angle *β*. From symmetrical point of view, one can predict that in the case of permanent rotation, an expected curve will be close to that at the angle *β* = *π*/4. However, Fig. 5 shows that the curves are slightly shifted down from the mean region in the complete interval between *β* = 0 and *β* = *π*. This leads to some shift of the mathematical expectation for the average |*V*_0_|. As a result, the value appears around angle *β* = *π*/5; the corresponding (smooth) curve is added to Fig. 8a.

## Biological interpretation

After getting into the first contact with the surface of surrounding objects, such as stones, other plants, soil, or potential dispersal agents, the fruit may experience an external force caused by wind, substrate vibrations, touch, etc. The results of our numerical modeling demonstrate that this force, if it is higher than some critical value, can either enhance contact area, when applied in one direction, or reduce contact area (and sequentially, adhesion) and even remove the fruit from the surface, when applied in another direction. If the force is lower than critical value, the contact (and sequentially, adhesion) will be almost always enhanced.

As it is mentioned in the previous paragraph, the result of this force action is angle-dependant. If the angle is situated in the way that it tends to rotate the fruit in an opposite direction relatively to the location of the majority of other adhesive glands, the chance to continue rotation and remove the fruit from the substrate is rather high. If the angle is situated exactly in the opposite direction, the chance for the fruit to be removed is low and the chance to enhance contact/adhesion is high. The intermediate angles would effect in an intermediate result with different degrees of probability.

However, in the reality, the external force is seldom oriented to one particular direction. It usually changes its direction, and the direction, in turn, can change the force rate/speed. The question appears how the changeability of the external force influences the fruit anchoring to the substrate. We found that if the external force changes its direction at the times corresponding to those of the fruit rotation, then in spite of non-constant force directionality, the fruit in contact will behave stably and sometimes even enhance its probability to adhere firmly to the substrate with the maximum number of its apical adhesive glands.

From the evolutionary point of view, the question about an optimal number of apical adhesive glands is probably one of the most important for biology of the plant. In the representatives of the genus *Commicarpus*, the typical number of the glands is 5 (one circle of glands) – 10 (two circles). The results of our simulation show that having only one or two contacts might be rather critical to maintain the contact under action of the external force. However with three contacts, the self-stabilization mechanism, although still rather weak, will take place. Furthermore, we did not obtain much stronger enhancement of contact formation in the fruits with more than five individual apical contacts. This particular geometry of adhesive contacts distribution at the circumference of the pyramid base reaches its saturation approximately at five individual contacts. In other words, more than five apical glands would be redundant for the effects discussed in this paper.

Natural substrates, which *Commicarpus* fruits usually adhere to, are not smooth and flat, but rather strongly corrugated. From our field observations, we concluded that the fruits adhere well to rough surfaces. Furthermore, the results of numerical simulation demonstrate that an increase of the roughness amplitude leads to an increase of adhesive ability of the fruit, because on the rough surface, the action of the external force will be always redirected to the direction, which almost never corresponds to the “bad” angle. That is why, this situation will effect in a stronger anchoring of the fruit to the 3D surface due to the recruiting of a higher number of individual apical glands in contact.

In summary, the specific geometry of *Commicarpus* fruits with the 5 + adhesive glands situated apically at the perimeter of the pyramid base represents an adaptation to enhance the number of glands in contact after initial adhesive contact formation with just one individual gland. Such an adhesive gland distribution increases adhesion to rough substrates and under action of the external force with changeable direction. More than three glands are sufficient for the occurrence of these effects. This geometrical adaptation, in combination with the properties of the glue itself, makes the fruit to the self-enhancing adhesive system capable of strong adhesion force generation after the formation of the first discrete adhesive contact. All these features contribute to the success of the epizoochorous fruit dispersal and to the fruit anchoring and stabilization between the stones or/and in soil.

## Electronic supplementary material

Supplementary Movies S1

Supplementary Movies S2

Supplementary Movies S3

Supplementary Movies S4

Supplementary Movies S5

Supplementary Information
