# Supplementary material for: Radial arrangement of apical adhesive sites promotes contact self-alignment of fruits in Commicarpus plants (Nyctaginaceae)
Source: Sci Rep. 2017 Sep 8;7:10956. doi: 10.1038/s41598-017-10567-9 (PMC5591214; doi:10.1038/s41598-017-10567-9)
Supplement: Supplementary file 6 — Supplementary Information [file 41598_2017_10567_MOESM6_ESM.doc]

Radial arrangement of apical adhesive sites promotes contact self-alignment of fruits in *Commicarpus* plants (Nyctaginaceae)

**Alexander E. Filippov, Elena V. Gorb and Stanislav N. Gorb**

**Supplementary Information**

**Supplementary Movies S1, S2, S3 and S4.** Dynamics of the attachment processes for the same realizations in the cases of the surface with the critical amplitude of roughness $A_{crit}\approx0.5$, angle $\beta=\pi/4$, external force $F_{ext}=0.25$, and varying adhesive points number of $N=$2,3,5 and 7, respectively. The difference in the rates of motion is clearly seen. The systems with $N=$5 and 7 (Supplementary Movies S3 and S4, respectively) move almost identically. After a relatively short transient period, they adjust and adhere to the surface. The systems with$N=$2 and 3 (Supplementary Movies S1 and S2, respectively) never stop at given parameters, but they move with very different velocities.

**Supplementary Movie S5.** Recorded at the same parameters as the Supplementary Movie S3, but for the over-critical force $F_{ext}=1.0$. It is seen from the movie, how the system may potentially continue its rotation and motion after initial attachment to the surface.
